# Vomiting and risk of endotracheal intubation related to preoperative doxycycline use for dilation and evacuation

**DOI:** 10.1016/j.contraception.2022.06.002

**Published:** 2022-06-17

**Authors:** Madeleine E. Weiss, Laura A Potter, Rabia Kamboj, Matthew D. Ponzini, Machelle D Wilson, Melody Y Hou

**Affiliations:** a University of California, Davis School of Medicine; Sacramento, CA, United States; b Kansas City University College of Osteopathic Medicine; Kansas City, MI, United States; c Department of Public Health Sciences, University of California, Davis, Sacramento CA, United States; d Department of Obstetrics and Gynecology, Family Planning, University of California, Davis, Sacramento CA, United States

**Keywords:** Doxycycline, Dilation and evacuation, Monitored anesthesia care, Vomiting

## Abstract

**Objective:**

To describe the rate of vomiting from oral doxycycline 200 mg given the night before second trimester dilation and evacuation (D&E), proportion of anesthesia modalities, and anesthetic complications.

**Study Design:**

We conducted a single-institution retrospective cohort study of patients presenting for second trimester D&E (14–0/7 to 23–6/7 weeks gestation) July 1, 2019-June30, 2020 following their scheduled preoperative visit as identified by billing codes. We recorded vomiting within 30 minutes of ingestion, anesthetic modality, and anesthetic complications. We tested for associations using chi-square or Fisher’s exact test for categorical variables and Wilcoxon-rank sum for non-normal numeric variables.

**Results:**

We reviewed 702 charts, of which 461 (66%) met inclusion criteria and 420 (60%) took doxycycline as prescribed. Of those who took doxycycline as prescribed, 30 (7.14%) reported vomiting within 30 minutes of ingestion. Nulliparity, primigravida and age less than 30 were significantly associated with vomiting (*p* = 0.005, *p* < 0.001 and *p* = 0.03, respectively), but gestational age (*p* = 0.53), BMI (*p* = 0.93), and gastrointestinal conditions (*p* > 0.99) were not. Only gravidity (*p* < 0.001) and parity (*p* = 0.01) remained significant in each of their respective multivariate models. None of the 10 patients who received general endotracheal tube anesthesia (2.4%) had vomited from doxycycline preoperatively. We observed 5 (1.2%) anesthetic complications (postoperative nausea or vomiting, anaphylaxis, and aspiration) that occurred only in those without vomiting.

**Conclusions:**

Vomiting rates following doxycycline were lower than those previously published. We found no significant association between doxycycline-associated vomiting and increased need for general endotracheal tube anesthesia or anesthetic complications; however, our study is underpowered to draw further conclusions.

**Implications:**

The findings of this study are consistent with guidelines indicating deep sedation as an effective anesthetic modality with low complication rates. Nulliparous patients may benefit from administration of an antiemetic prior to doxycycline prophylaxis, but routine antiemetic use may not be necessary.

## 1. Introduction

The preferred modality of anesthesia for second trimester dilation and evacuation (D&E) is deep sedation without general endotracheal tube anesthesia (GETA), as intubation is associated with increased risk of complications including greater intraoperative blood loss [1]. Previous studies have shown that deep sedation for abortion has low complication rates ranging from 0% to 0.3% [2–6]. Nevertheless, perioperative pulmonary aspiration remains a concern of deep sedation anesthesia in pregnancy due to hormonal and anatomical changes [7]. Additional risk factors for aspiration include pre- or intraoperative vomiting and gastro-esophageal reflux disease (GERD) [8].

Oral doxycycline is a common prophylactic antibiotic for second trimester D&E procedures, but is associated with vomiting, which may increase the risk of needing GETA [9, 10]. Guidelines recommend doxycycline administration the night before D&E procedures rather than within two hours of surgery to reduce the rates of nausea and vomiting and thus a need for GETA [9, 11]. Doxycycline taken the night before D&E does not significantly alter antibiotic serum levels at time of procedure [9]. A recent study demonstrated that administering the antiemetic ondansetron 30 minutes prior to oral doxycycline the night before D&E decreased the risk of vomiting by two-thirds, with a statistically significant but clinically insignificant increase in doxycycline serum levels at the time of procedure [9]. Investigators did not report relative rates of different anesthesia modalities for patients with and without vomiting. However, the rate of vomiting among patients taking doxycycline without ondansetron was 50%, more than triple the rates previously reported [11].

We aimed to characterize our rates of vomiting within 30 minutes of oral doxycycline 200 mg ingestion the night before D&E, differences in deep sedation versus GETA anesthetic modalities, and anesthetic complications.

## 2. Methods

We conducted a retrospective cohort study of patients with a second trimester (14 0/7 to 23 6/7 weeks gestation) D&E procedure at the University of California, Davis Medical Center (UCDMC) between July 1, 2019 and June 30, 2020. The University of California, Davis Institutional Review Board classified this study as exempt. We identified patients in the electronic medical record using a billing records query for Current Procedural Terminology code 59841.

All patients analyzed in this study attended a scheduled preoperative outpatient visit the day before surgery, during which history, procedure description and consent, preoperative and postoperative expectations and precautions, contraception counseling, and osmotic cervical dilator insertion were completed. At UCDMC, the standard of care at this preoperative visit is to prescribe patients four tablets of doxycycline 100 mg for antibiotic prophylaxis, with instructions to take 2 tablets (200 mg) orally with food the evening before surgery, as well as ibuprofen and oxycodone as needed for pain control with cervical dilators. When patients arrive on the day of surgery, the surgical team assesses antibiotic intake and vomiting. Those who did not take their antibiotic as prescribed or who reported vomiting within 30 minutes of taking doxycycline routinely receive intravenous doxycycline 100 mg prior to entering the operating room. Patients with vomiting more than 30 minutes after taking doxycycline do not receive additional antibiotic prophylaxis. Postoperatively, we instruct patients to take the remaining 200 mg oral doxycycline with their first meal after their procedure. For patients at 20 weeks gestation or greater, we also prescribe methergine for bleeding prophylaxis for 48 hours. All patients receive postoperative paperwork with these dosing instructions along with return precautions, recovery expectations and medication risks.

We excluded patients who did not have a documented outpatient preoperative visit at UCDMC prior to their D&E procedure; those without documented preoperative medications, surgical procedure, or anesthesia records; and those who were not prescribed doxycycline for antibiotic prophylaxis. For those patients with more than one D&E procedure within the study period, we included the first event only.

We collected patient characteristics such as age, body mass index (BMI), race, ethnicity, gravidity, parity, history of cesarean delivery, and history of gastrointestinal (GI) conditions. We defined a history of GI conditions as documentation of any GI-related medical and surgical diagnoses self-disclosed by the patient during the preoperative visit or in the anesthesiology preoperative assessment. The anesthesiology assessment includes a specific inquiry of gastroesophageal reflux disease. We recorded preoperative opioid use and antiemetic prescription, antibiotic prophylaxis prescription and intake, occurrence of vomiting within 30 minutes of doxycycline ingestion, and administration of additional antibiotics when vomiting occurred. Additionally, we assessed anesthetic modality and anesthetic complications including postoperative nausea and vomiting, anaphylaxis, and aspiration during anesthesia induction. We recorded data using Research Electronic Data Capture (REDCap) [12].

We examined covariates for associations with vomiting, GETA use, and anesthetic complications using chi-square or Fisher’s exact test for categorical variables and Wilcoxon-rank sum for any non-normal numeric variables. We also fit 2 multivariable models for vomiting with variables that were significantly associated with our outcome in the univariate analysis, including history of GI conditions, age at D&E, gestational age, and either gravidity or parity, and we employed a backward iterative selection procedure in which only those variables that remained significant were retained in the model. Separate multivariable models, one using gravidity and the other parity, were indicated as these two variables are highly correlated with each other and thus cannot be included in the same model. We used SAS^®^ software version 9.4 for Windows^®^ and set our significance threshold at *p* < 0.05 in all analyses.

## 3. Results

**Fig. 1.**
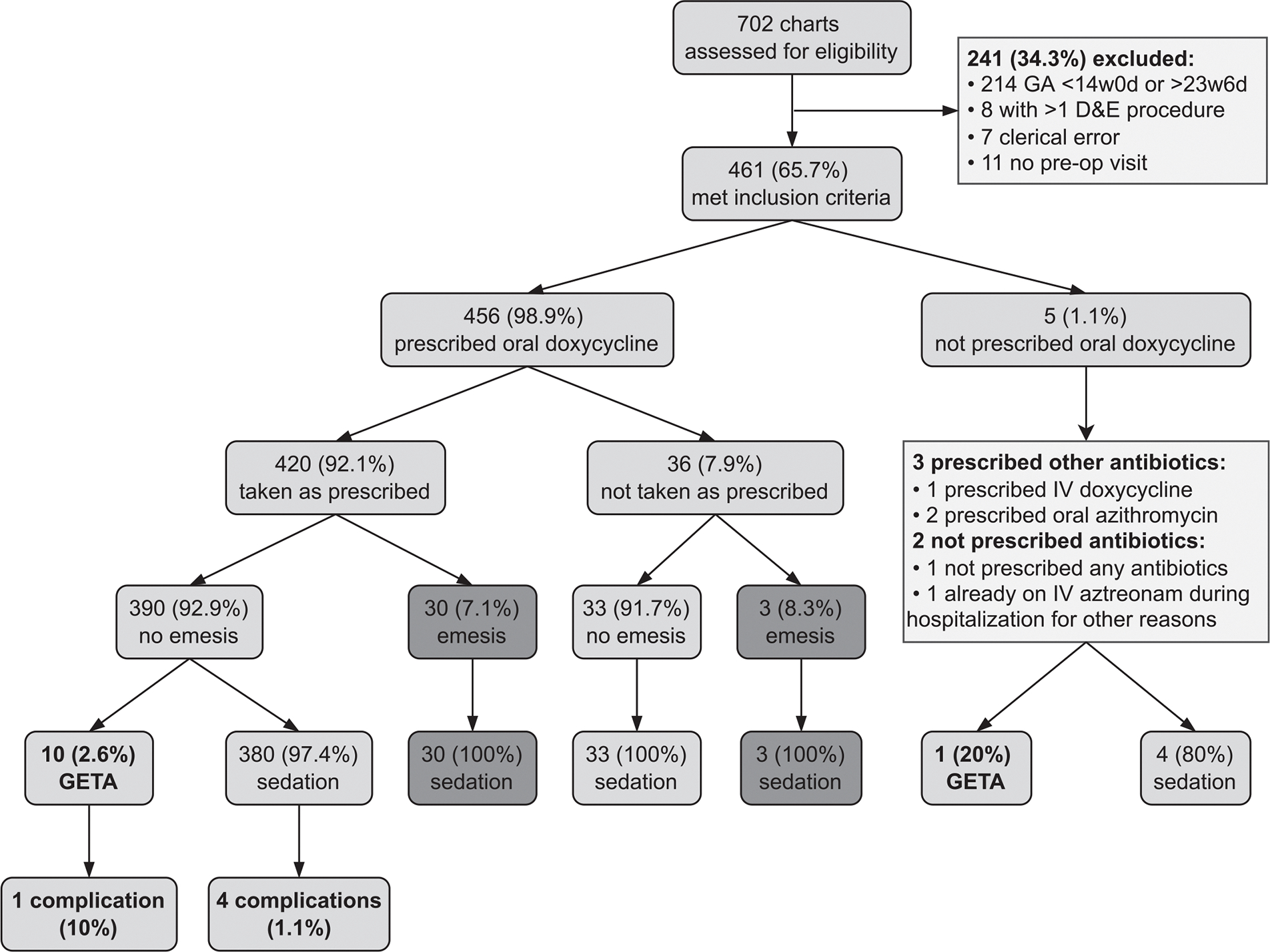
Flow diagram of patients undergoing second-trimester surgical abortion at UCDMC from July 1, 2019 to June 30, 2020. Unless otherwise reported, zero anesthetic complications occurred. GA = gestational age; w = weeks; d = days; D&E = dilation and evacuation; pre-op = preoperative; IV = intravenous; GETA = general endotracheal tube anesthesia.

We reviewed 702 instances of second trimester D&E during the 1-year evaluation period, out of which 456 patients met inclusion criteria and 420 took doxycycline as prescribed (Fig. 1). We identified no demographic differences between those who took doxycycline and those who did not take doxycycline as prescribed. Of the 36 patients who did not take oral doxycycline as prescribed, 3 (8.3%) reported vomiting and 4 (11.1%) noted medication nonadherence such as misunderstanding of dosing instructions. Another two patients cited difficulty swallowing pills and inability to pick up medication from the pharmacy. The remaining 27 patients did not have reasons documented for not taking the doxycycline as prescribed. Characteristics of those who took doxycycline as prescribed are listed in Table 1. Of the patients who took doxycycline as prescribed, 30/420 (7.14%) reported vomiting within 30 minutes, all of whom subsequently received deep sedation without intubation. Of the remaining 390 who did not vomit within 30 minutes of taking their antibiotics as prescribed, 10 received GETA and the remainder received deep sedation. The vomiting rate among those who took oral doxycycline as prescribed was not significantly different from that of the patients who did not (30/420, 7.14% and 3/36, 8.33%, respectively; *p* = 0.74).

Univariate analyses revealed that patients under 30 years of age were more likely to report vomiting than patients over 30 (*p* = 0.02). Primigravid patients had a higher risk of vomiting compared to multigravid patients (16/74 (21.6%) and 14/346 (4.05%), respectively; *p* < 0.001). Similarly, nulliparity was associated with a higher risk of vomiting compared to multiparity (18/133 (13.53%) and 12/287 (4.18%), respectively; *p* = 0.005). Rates of vomiting by BMI category (<25, 25–29.9, 30–39.9, >40) did not differ (*p* = 0.93). We found no association between gestational age (*p* = 0.53), history of GI conditions (*p* > 0.99), or history of cesarean delivery (*p* = 0.38) and vomiting. Eight of 420 patients (1.9%) had an antiemetic prescribed during their preoperative visit and 205/413 patients (49.6%) reported taking a prescribed preoperative opioid. Neither were associated with vomiting (*p* = 0.47 and 0.85, respectively). In each of their respective multivariable models, only gravidity (*p* < 0.001) and parity (*p* = 0.01) remained significant after backward selection.

We identified 5 instances of anesthetic complications, including postoperative nausea or vomiting (n = 3), anaphylaxis (n = 1), and aspiration (n = 1). Neither the occurrence of anesthetic complications (*p* > 0.99) nor the modality of anesthesia (*p* > 0.99) was associated with post-antibiotic vomiting. Furthermore, the modality of anesthesia did not correlate with anesthetic complications (*p* = 0.11).

## 4. Discussion

Our vomiting rate of 7.14% from prophylactic oral doxycycline for surgical abortion is lower than rates of 15% and 28.3% previously reported in other studies [7,9]. We did not find an increased rate of GETA among those with vomiting, which is reassuring since GETA is associated with increased intraoperative blood loss, prolonged recovery, and delayed arousal [1]. Furthermore GETA is significantly more expensive than deep sedation, also called monitored anesthesia care (MAC), with one study indicating a cost of $61.74/case for GETA versus $26.27/case for MAC [13]. Doxycycline-associated vomiting did not appear to change choice of anesthetic modality at UCDMC, where deep sedation is standard of care for D&E. We also did not find an increased rate of anesthetic complications among those who vomited following doxycycline and subsequently received deep sedation. Though our study is inadequately powered to draw a conclusion regarding anesthetic complications, our finding is consistent with the most recent Society of Family Planning guidelines on anesthesia in surgical abortion which endorse deep sedation as an effective anesthetic modality with low complication rates [14].

While not associated with increased risk of anesthetic complications, primigravid and nulliparous patients were more likely to vomit following doxycycline, which is consistent with previous research documenting an association between low gravidity and nulliparity and both hyperemesis gravidarum and early pregnancy vomiting [15,16]. Primigravid and nulliparous patients may benefit from pre-administration of an antiemetic, such as ondansetron, 30 minutes before doxycycline intake for possible side effect relief [9].

Strengths of our study are the large sample size and examination of a common D&E preoperative doxycycline prophylaxis regimen. Limitations of the study include its retrospective nature, which restricts our ability to understand what other undocumented factors may be associated with vomiting or receipt of deep sedation versus GETA such as preexisting gestational nausea and vomiting. Since patients self-report whether they took doxycycline as prescribed and whether they experienced vomiting within 30 minutes thereof, our study may be subject to recall bias. Although we had no differences in anesthetic modality or complications due to vomiting, our anesthesiologists are very comfortable with deep sedation for our procedures which may limit generalizability. Overall, this study showed low rates of vomiting after doxycycline 200 mg taken orally the night before second trimester D&E. Additionally, this study did not demonstrate that doxycycline-associated vomiting is associated with increased need for GETA or with anesthetic complications, but lack of power limits our ability to draw conclusions.

## Figures and Tables

**Table 1:** Characteristics of patients undergoing second-trimester surgical abortion at UCDMC from July 1, 2019 to June 30, 2020 who took oral doxycycline as prescribed (n = 420)

Characteristic	n (%) or Median (Range)

Age (yrs)	29 (13, 46)
Gestational age (wks)	19.43 (14, 23.86)
Gravidity	
1	74 (17.62)
2	66 (15.71)
3 +	280 (66.67)
Parity	
0	133 (31.67)
1	101 (24.05)
2	87 (20.71)
3+	99 (23.57)
Race	
White	198 (47.14)
Black/African-American	78 (18.57)
Asian	36 (8.57)
American-Indian/Alaska native	7 (1.67)
Native Hawaiian/Pacific Islander	4 (0.95)
More than one race	16 (3.81)
Unknown/not reported/other	84 (19.29)
Ethnicity	
Hispanic or Latino	125 (29.76)
Not Hispanic or Latino	287 (68.33)
Unknown/not reported	8 (1.90)
BMI	
Underweight [< 18.5]	4 (0.95)
Normal (18.5–25)	117 (27.86)
Overweight [25–30)	132 (31.43)
Obese [30–40)	125 (29.76)
Morbidly obese [>40]	41 (9.76)
Unknown	1 (0.24)
History of gastrointestinal conditions*	
Yes	57 (13.57)
No	363 (86.43)
Previous cesarean delivery	
Yes	113 (26.90)
No	307 (73.10)
Prescribed preoperative antiemetic	
Yes	8 (1.90)
No	412 (98.10)
Took preoperative opioid	
Yes	205 (48.8)
No	208 (49.5)
Unknown	7 (1.7)

* Includes gastroesophageal reflux disease (GERD), Hepatitis C, alcoholic cirrhosis, bulimia, cholelithiasis, cholecystectomy, gastric bypass or sleeve, and esophageal variceal banding.
